# Emesis in a Horse

**Published:** 1884-01

**Authors:** William Herbert Lowe

**Affiliations:** Little Falls. N. J.


### III.—EMESIS IN A HORSE.

BY WILLIAM HERBERT LOWE, D.V.S.

As vomiting in the horse is of seldom or rare occurrence, and recovery therefrom still more rare, I propose to report briefly a case which came under my notice in Brooklyn, and which was treated by Dr. J. F. Mustoe.

The patient was a bay gelding, aged, 15-2 hands, and had a good record as a trotter. The history given by the owner was as follows: The horse was known to have vomited on two previous occasions. Shortly after the vomiting each time he regained his usual health. Prior to present attack the horse was in good health, showed no symptoms of colic, and was driven moderately the morning before his illness. Was eating his noon food when the attendant noticed that he was indisposed and vomited. At time of examination pressure over the trachea caused a regurgitation of liquid and food, both from the mouth and the nostrils. The animal showed symptoms of much distress and pain, sweated profusely, breathing rapid and labored, pulse feeble and irregular, and symptoms very similar to those of rupture of the stomach. But for the history rupture of the stomach would have been suspected and an unfavorable prognosis given. It was advised that the animal be kept quiet and without food until the following morning. Small and frequently repeated doses of nux vomica tr. were given.

Next morning the horse was apparently, if not really, well. Three days afterwards he was brought to Dr. Mustoe with the complaint that he would not eat. On examination, the mucous membranes were found to be of a peculiar yellow color with symptoms of jaundice. Appropriate remedies were administered. In four days the horse was discharged his appetite having returned and being in good health. Since which time he has been driven frequently and has manifested no unfavorable symptoms.

LITTLE FALLS. N. J.

